# Dynamics of total volume of pancreatic α‐ and β ‐cells under the influence sulfonylureas and their combination with dipeptidyl peptidase‐4 inhibitors

**DOI:** 10.1002/edm2.238

**Published:** 2021-03-18

**Authors:** Tuchina Taisiia Pavlovna, Skotnikova Kseniia Petrovna, Vtorushina Anna Anatolievna, Uskov Ivan Sergeyevich, Rogoza Olga Vladimirovna, Grozov Roman Viktorovich, Babenko Alina Yurievna, Galagudza Mikhail Mikhailovich

**Affiliations:** ^1^ Almazov National Medical Research Centre Saint Petersburg Russia

**Keywords:** dipeptidyl peptidase‐4 inhibitors, sulfonylurea, type 2 diabetes

## Abstract

**Objective:**

Sulfonylureas and dipeptidyl peptidase‐4 inhibitors have a multidirectional effect on pancreatic cells. We aimed to evaluate the effects of these drugs on β‐ and α‐cells in rats aged 12 months with type 2 diabetes mellitus when administered in combination with various sulfonylureas.

**Methods:**

Streptozotocin‐nicotinamide induced type 2 diabetes mellitus was induced in rats aged 12 months. Animals received the sulfonylureas gliclazide or glibenclamide for 24 weeks alone or in combination with the dipeptidyl peptidase‐4 inhibitor vildagliptin or vildagliptin monotherapy. Blood glucose and animal weights were measured before and during the experiment. The oral glucose tolerance test was conducted before therapy initiation. Immunohistochemical analyses were conducted after the end of the experiment using glucagon and insulin antibodies. The total volumes of α and β cells were estimated in relation to the volume of the pancreatic islets.

**Results:**

The total volumes of β‐cells did not differ between the sulfonylurea group and the untreated type 2 diabetes mellitus group. The addition of dipeptidyl peptidase‐4 inhibitors to sulfonylureas normalized the total volumes of β cells. The total volumes of α‐cells in the gliclazide group and combination of gliclazide with dipeptidyl peptidase‐4 inhibitor was comparable to that in the control group of intact animals, in contrast with the glibenclamide group.

**Conclusion:**

The combination of vildagliptin to both sulfonylureas contributed to the normalisation of β‐cells number. Normalisation of the total volumes of α‐cells was observed with gliclazide therapy and combination of gliclazide with dipeptidyl peptidase‐4 inhibitor.

## Graphical abstract

The study demonstrates the features of the pancreatic islets cells of ageing rats with diabetes mellitus type 2 with sulfonylureas and dipeptidyl peptidase‐4 inhibitor treated, as well as a combination of these drugs.

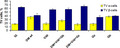

## 1 OBJECTIVE

Currently, the number of patients with type 2 diabetes mellitus (T2DM) is steadily increasing, and the search for an optimal T2DM treatment remains relevant. Globally, there are 11 classes of antidiabetic drugs to treat T2DM; however, it is not always possible to establish long‐term control of glycemia. One probable reason is the gradual loss of β‐cells that progresses with the duration of T2DM.^1^ It is believed that the physiological number of β‐cells is maintained by a balance between apoptosis and proliferation.^2^ The most pronounced apoptosis and progressive loss of β‐cells are observed in older patients.^3^ Sulfonylureas are one of the longest‐used drugs and are often prescribed in the Russian Federation and globally.

However, studies regarding the effect of sulfonylureas on pancreatic islet morphology have been largely limited to estimating the number of β‐cells only, and their results are ambiguous due to a variety of study conditions that have been used. Also, publications suggest that sulfonylurea therapy can cause β‐cell dysfunction and apoptosis.^4^

It has also been shown that the effect on the pancreas depends on the choice of sulfonylureas. In particular, it was reported that the apoptosis‐stimulating effect of glibenclamide on cultured cells *in vivo* was significantly more pronounced than the effect of glimepiride, gliclazide did not stimulate apoptosis.^4^, ^5^ The same data were obtained with gliclazide by other researchers using Goto‐Kakisaki rats.^5^

There are isolated data showing that β‐cell deficiency develops during sulfonylurea therapy that leads to an increase in the glucagon response and α‐cell hyperplasia through the mechanism of paracrine regulation.^5^ Other studies showed that glibenclamide did not violate the morphology of the pancreas and the distribution of α and β cells compared with healthy control. However, the duration of therapy was limited to a maximum of 10–12 weeks in these studies.^6^

Gliclazide structure differs from other sulfonylureas by the presence of the aminoazabicyclo(3.3.0)octane ring that provides it with pleiotropic antioxidant properties. Unlike other sulfonylureas, gliclazide reversibly binds to a receptor on β cells that reduces the risk of depletion of the functional reserve, suggesting that the treatment with glyclazide is associated with a lower incidence of β‐cells apoptosis.

The effect of various sulfonylureas on the morphology of pancreatic cells has not yet been fully elucidated. Another commonly prescribed group of antidiabetic drugs are dipeptidyl peptidase‐4 inhibitors (DPP4i). These drugs tend to increase the replication of β cells and inhibit their apoptosis.^7^ In addition, DPP4i can restore the physiological regulation of glucagon levels by α cells. An increase in the proliferation of β and α cells was noted in individual studies.^7^ However, our earlier studies did not confirm the conclusion of an increased proliferation of pancreatic cells.^8^ Taking into account the multi‐factorial effect of the above drugs on pancreatic cells, it would be interesting to evaluate the effects of drug‐combination therapy on β and α cells. It is known that gliclazide's mechanism of action is fundamentally different from other sulfonylureas. Unlike other sulfonylureas, it interacts with the β‐cell SUR1 receptor, does not bind to the Epac2 factor, and causes an excessive release of insulin. Accordingly, unlike other sulfonylureas, it acts only on the SUR1 receptor without affecting other secretion‐activation pathways (cAMP signalling pathway in β cells and Epac2A protein) that are involved in the implementation of incretins.

Hence effect of gliclazide on the volume of β and α cells can significantly differ from the effects of other sulfonylureas in combinations with DPP4i. We embarked on an experimental study to assess the morphological composition of pancreatic islets in T2DM rats aged 12 months experimental model receiving sulfonylurea therapy and a combination of sulfonylureas with DPP4i. Such experimental studies make it possible to evaluate the effects of these drugs under the conditions that are difficult to implement in clinical studies: long duration of continuous therapy and a thorough morphological assessment and immunohistochemistry (IHC) of the pancreas.

## 2 MATERIALS AND METHODS

### 2.1 Animals

60 Wistar rats aged 12 months were used in this study. The animals were maintained under a 12/12‐h light/dark cycle with free access to food and water, unless otherwise specified.

### 2.2 T2DM induction

A streptozotocin‐nicotinamide‐induced T2DM model was generated in 50 random rats using the method reported by Bayrasheva et al.^9^ The remaining 10 animals were in the intact control group. We used a high‐fat diet for this model to create the metabolic changes associated with T2DM.^9^ These 50 animals were transferred for 5 weeks to a high‐fat diet with the addition of beef fat to the standard feed. The total caloric content of the food was 450 kcal/100 g (20%–fat, 48%–carbohydrates and 20%–protein).^9^ These rats were without food for 12 hours after a period of high‐fat diet. We injected a nicotinamide solution (230 mg/kg) intraperitoneal, then 15 minutes later we injected a streptozotocin solution (65 mg/kg) intraperitoneal.^10^ Nicotinamide (Herba Hemosan, Austria) was previously dissolved in 0.9% sodium chloride solution. Streptozotocin (Sigma Aldrich) was dissolved in a freshly prepared sodium citrate buffer.^11^ We injected 0.9% sodium chloride solution to 10 intact animals during the simulation of T2DM.

The development of experimental type 2 diabetes in rats is confirmed by monitoring the level of fasting glycemia (on an empty stomach at 9:00 am, after 8–12 h of starvation), followed by the oral glucose tolerance test (OGTT) with oral or parenteral administration of glucose solution.^12^

Diagnostic criteria for T2DM included fasting blood glucose level 9–14 mmol/L (OneTouch Select glucometer, LifeScan Inc.) and more than 2‐fold increase in the calculated area under the glucose curve based on OGTT results in comparison with intact animals.^12^

Experimental studies were conducted at the Federal State Budget Scientific Institution ‘Institute of Experimental Medicine’ in cooperation with the staff of the Laboratory of Chemistry and Pharmacology of Medicine, following the ‘Guidelines for the Care and Use of Laboratory Animals’ and ‘A guide to the experimental (preclinical) study of new pharmacological substances’, in accordance with the principles of humanity, European Directives (86/609/EEC) and Helsinki Declaration.

### 2.3 Glucose control and OGTT

The rats fasted for 12 h were given a bolus of glucose (2 g/kg). Blood glucose was measured in samples obtained from the tail vein at 0, 30, 60, 90 and 120 min after glucose administration using a glucometer.

We monitored blood glucose level on an empty stomach at 9:00 am, after 8–12 h of fasting every 2 weeks during the experiment.

### 2.4 Experimental design

The animals were included in the experiment at the age of 12 months. The blood glucose level and weights of the animals were determined five‐week after inducing the T2DM model; the OGTT was also performed at that time.

The rats with confirmed T2DM received treatment for 24 weeks according to the following therapy groups (5 animals each), sorting by therapy groups was random. There was also a group of intact animals (10 animals) and group T2DM without therapy (10 animals).

1. Intact group ‐ control group without T2DM and without therapy.
2. T2DM without therapy.
3. T2DM + vildagliptin (1.5 mg per kg).
4. T2DM + vildagliptin (1.5 mg per kg) + glibenclamide (0.6 mg per kg).
5. T2DM + vildagliptin (1.5 mg per kg) + gliclazide (0.75 mg per kg).
6. T2DM + gliclazide (0.75 mg per kg).
7. T2DM + glibenclamide (0.6 mg per kg).

Blood glucose was determined at 4, 10, and 24 weeks after experiment initiation. The weight of animals was measured at week 24 of the study. Pancreatic tissue sampling and IHC were performed after the end of the experiment.

### 2.5 Morphological study

Pancreatic preparations were made after the end of the experiment. The departments of the pancreas differ in the number of β and α cells. In our study, we used pancreatic tissue from the spleen segment of the rat pancreas.^13^

Pancreatic tissue was placed in a 10% buffered formalin solution for 24–48 hours. The processed tissue material was embedded in histological paraffin, sectioned on a microtome Leica EM UC7 (Austria), and fixed on histological glasses with an adhesive coating. The microscopic structure of the pancreas tissue was evaluated after staining with haematoxylin‐eosin. The haematoxylin‐eosin sections were viewed on an inverted microscope Leica Aperio AT2(Austria).

### 2.6 IHC

IHC was performed using glucagon and insulin antibodies.

Glucagon antibodies (Abcam ab10988, monoclonal mice) were used in a 1:10,000 dilution, incubated with primary antibodies for 60 min at room temperature, and unmasked in a pH 6 buffer at 98°C for 30 min (Thermo detection system; Steiner Thermo Autostainer 720).

Insulin antibodies (Abcam ab6995, monoclonal mice) were used in a 1:1000 dilution, incubated with primary antibodies for 60 min at room temperature, and unmasked with trypsin (Thermo) at 37°C for 10 min. (Abcam detection system was used in a manual setting).

### 2.7 Microphotographs

Photos of micro‐preparations in IHC were made using Leica equipment and analysed using Photoshop.

### 2.8 Evaluation of results

The total volume (TV) of α and β cells were estimated relative to the area of the pancreatic islet. 10–30 islets were found on each histological preparation. Average values of cell TV were used for each group.

### 2.9 Statistical analysis

Statistical analyses were performed by a biomedical statistician using the IBM SPSS Statistics 23 programme (SPSS Inc.). Data were presented as means ± SD (M ± m). To evaluate the differences between dependent samples, the non‐parametric Wilcoxon test was used; the Mann‐Whitney test was used to evaluate the reliability of the differences between independent variables. *P*‐values < 0.05 were considered statistically significant.

## 3 RESULTS

### 3.1 Bodyweight

No significant differences in animal weights were detected initially and after 24 weeks of observation. The weight of animals of different groups did not differ significantly.

### 3.2 Glucose control and OGTT

**FIGURE 1:**
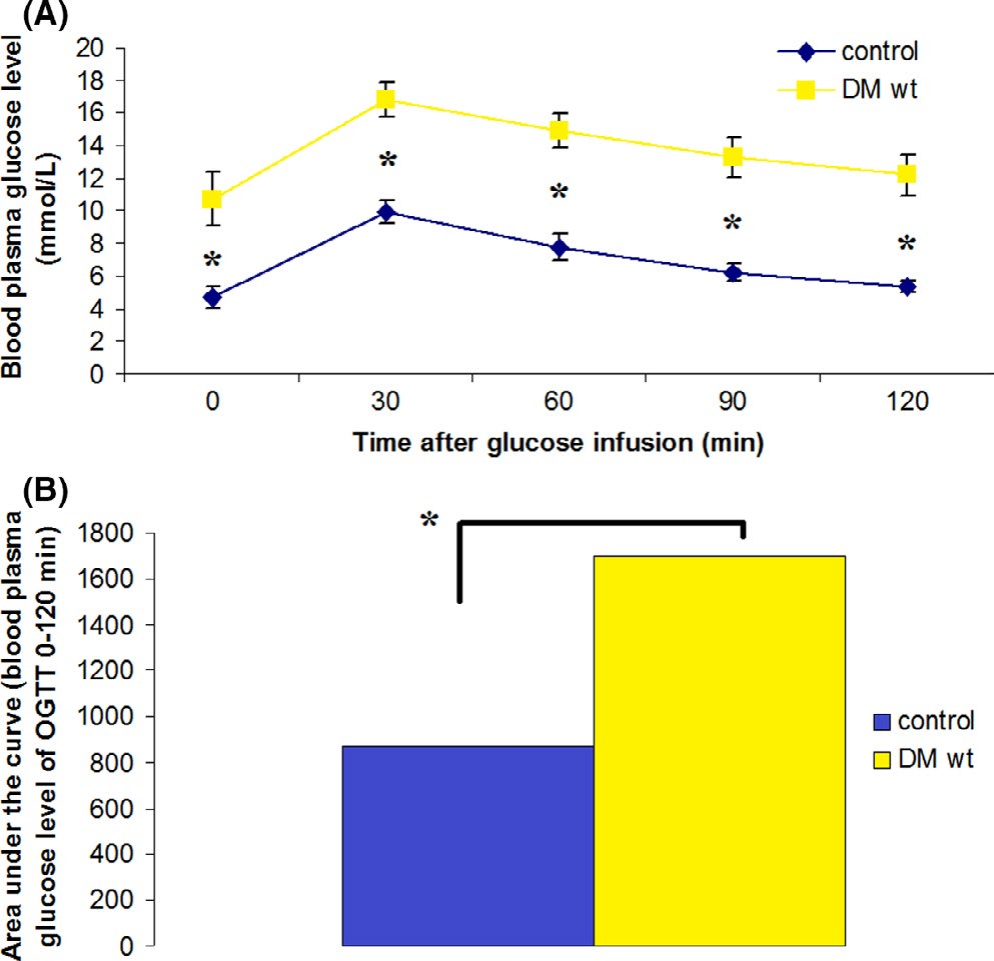
OGTT results. A, Blood plasma glucose level, based on the results of OGTT in the control group (IG) and in the groups of T2DM (DM wt). * *p* < 0.05. B, Area under the curve by results OGTT in the control group (IG) and in the groups of T2DM (DM wt). * *p* < 0.05

The Figure 1A,B shows the results of OGTT performed 1 week after intraperitoneal consecutive injections of nicotinamide and streptozotocin in 50 rats or saline solution in 10 rats of the control group of intact animals. Severe hyperglycemia (above 20.0 mmol/l) developed in 10% of animals after injections of nicotinamide and streptozotocin, these animals were excluded from the experiment. The level of glycemia during OGTT did not correspond to the level of glycemia typical for T2DM in 20% of rats after injections of nicotinamide and streptozotocin (these animals were excluded from the experiment). A significant difference in glycemia level between animals with type 2 diabetes and intact animals was observed at all‐time points (0, 30, 60 and 120 min) (*p* < 0.05) (Figure 1A). Subsequent calculation of the area under the glucose curve (Figure 1B) confirmed the development of T2DM, according to the criteria of Peterson G. et al. in 70% of the rats that we modelled T2DM.[60].

**FIGURE 2:**
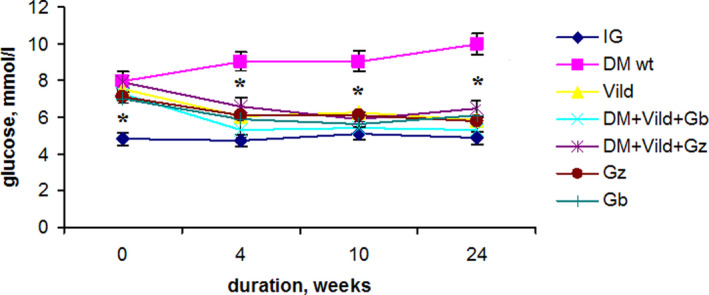
Blood glucose level during the experiment (on an empty stomach at 9:00 am, after 8–12 hours of starvation). Treatment groups: IG: control group of healthy animals (n = 10); DM wt: T2DM without therapy (n = 10); Vild: T2DM + vildagliptin (n = 5); DM + Vild + Gb: T2DM + vildagliptin + glibenclamide (n = 5); DM + Vild + Gz: T2DM + vildagliptin + gliclazide (n = 5); Gz: T2DM + gliclazide (n = 5); Gb: T2DM + glibenclamide (n = 5). *p < 0.05 Between IG and all other groups at the beginning of the experiment. Between DM wt and all other groups at the 4, 10, 24 weeks of the experiment

Blood glucose level in rats with T2DM remained stable and elevated until the end of the experiment at week 24 (Figure 2).

### 3.3 ICH results

As stated above, the total volume (TV) of α and β cells were estimated relative to the area of the pancreatic islet.

The TV of β cells was significantly reduced in the T2DM without treatment group (41,5%) compared with that in intact animal group (63,6%) (*p* < 0.05) but the total volume of α cells was increased (T2DM without treatment ‐ 37,1%, intact group ‐ 17,1%) (*p* < 0.05).

The TV of α cells did not change in the glibenclamide group (38,2%) compared with that in the T2DM without therapy group (37,1%) (*p* > 0.05). The decrease in the TV of α cells (*p* < 0.05) was observed in vildagliptin (18%), gliclazide (17,6%), and gliclazide +vildagliptin group (16,5%) compared with T2DM without therapy group, becoming comparable to the intact animal group (17,1%) (*p* > 0.05).

The TV of α cells in the glibenclamide +vildagliptin group (27,1%) was significantly lower than in the T2DM without therapy group (37,1%) but was significantly greater than in the intact group (17,1%) (*p* < 0.05).

**FIGURE 3:**
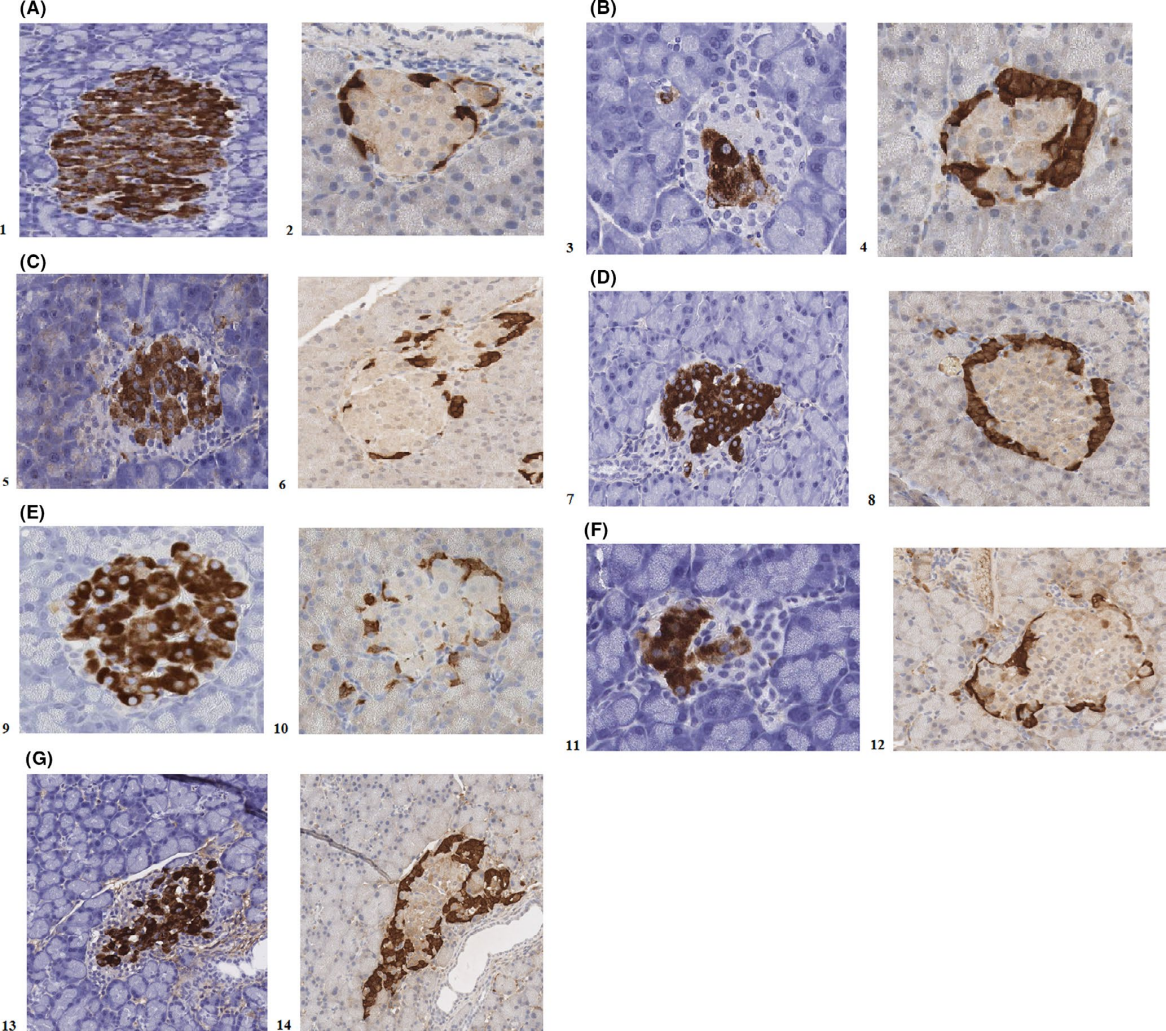
IHC microphotographs (20× magnification) of pancreatic islets after 24 weeks of therapy. A. 1. Intact group, insulin antibodies; 2. Intact group, glucagon antibodies; B. 3. T2DM without therapy group, insulin antibodies; 4. T2DM without therapy, glucagon antibodies; C. 5. T2DM + vildagliptin, insulin antibodies; 6. T2DM + vildagliptin, glucagon antibodies. D. 7. T2DM + vildagliptin + glibenclamide, insulin antibodies; 8. T2DM + vildagliptin + glibenclamide, glucagon antibodies; E. 9. T2DM + vildagliptin + gliclazide, insulin antibodies; 10. T2DM + vildagliptin + gliclazide, glucagon antibodies; F. 11. T2DM + gliclazide, insulin antibodies; 12. T2DM + gliclazide, glucagon antibodies; G. 13. T2DM + glibenclamide, insulin antibodies; 14. T2DM + glibenclamide, glucagon antibodies

**FIGURE 4:**
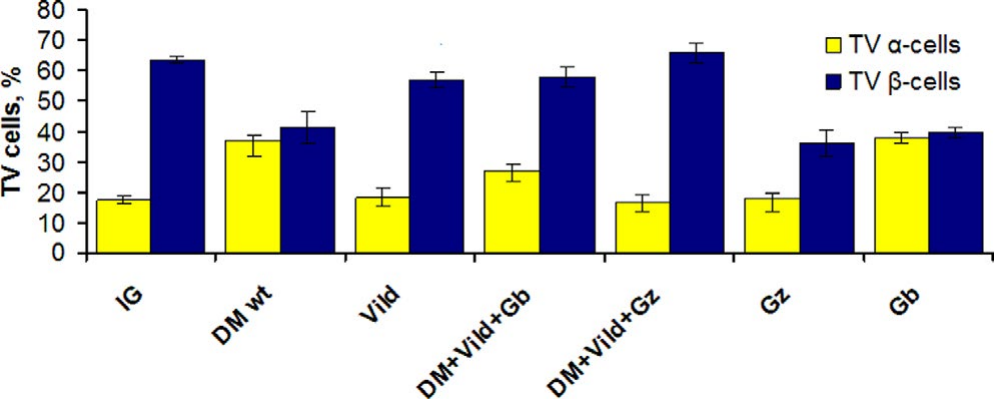
TV of α and β cells (%) of the pancreas in groups after 24 weeks of observation. IG ‐ healthy animals. DM wt: T2DM without therapy; Vild: T2DM + vildagliptin; DM + Vild + Gb: T2DM + vildagliptin + glibenclamide; DM + Vild + Gz: T2DM + vildagliptin + gliclazide; Gz: T2DM + gliclazide; Gb: T2DM + glibenclamide. *p* < 0.05 for TV of α cells in IG to DMwt, Gb groups. *p* < 0.05 for TV of β cells in IG to DMwt, Gb, Gz groups

The TV of β cells did not change in the sulfonylurea‐monotherapy groups (glibenclamide—39,9%, gliclazide—36,2%) compared with that in the T2DM without therapy group (41,5) (*p* > 0.05). The TV of β cells in the sulfonylurea +DPP4i combination (vildagliptin+glibenclamide—58,1%, vildagliptin+gliclazide ‐ 36,2%) and vildagliptin‐monotherapy groups (57%) were comparable to the intact animal group (63,3%) (*p* > 0.05). (Figures 3A‐G, 4).

## 4 DISCUSSION

Our data indicate that the ratio of α and β cells in rats aged 12 months (approximately 40 years in human years) with experimental T2DM corresponds to the ratio of pancreatic cells in patients with T2DM. Our model of T2DM maximally models to the clinical practice. Inclusion of animals at the age of 12 months allows us to assess the state of endocrinocytes at the age most typical for the development of T2DM. It is known that the development of T2DM is accompanied by a decrease in the TV of β cells and an increase in the TV of α cells.^14^ Possible causes of α‐cell hyperplasia and a decrease in the number of β cells in T2DM are being actively studied and several possible mechanisms are being considered. One of the mechanisms is chronic inflammation. The condition is characterized by hyperproduction of proinflammatory cytokines including IL‐6 that may be one of the regulators of the number and function of α cells. Hyperproduction of IL‐6 has been found to occur at the visceral‐obesity stage and can also be increased by a high‐fat diet. This is considered to be one of the mechanisms of endocrinocytes dysfunction in T2DM. We did not evaluate the level and expression of IL‐6 in this study but our experimental model did include a high‐fat diet; this enables us to assume that this mechanism also plays a role in the development of endocrinocytes dysfunction in our study.^15^

Another significant mechanism of α‐cell hyperplasia and decreased β‐cell number in our model may be the dedifferentiation of β cells into β‐cell progenitors. It occurs under the influence of hyperglycemia, high‐fat nutrition, and other factors. Dedifferentiation of β cells into β cell progenitors may lead to re‐differentiation into other types of pancreatic islet cells, in particular into α cells under the persistent influence of adverse factors. But there was no activation of proliferation processes in our study. This finding supports the role of re‐differentiation in these alterations. In addition, studies have reported that if the normal level of glycemia and weight are restored in individuals who recently developed T2DM (less than 6 years of duration), normalization of the cellular composition of the pancreatic islet occurs and remission of T2DM is possible.^16^, ^17^

According to the literature, administering sulfonylureas leads to a rapid functional depletion of β cells.^18^, ^19^ However, research on the effect of sulfonylureas on pancreatic cells suggests a different outcome (see our review of the literature above). Some researchers noted an increase in apoptosis by glibenclamide therapy but others did not.^18^, ^19^

Studies using gliclazide have not reported an increase in β‐cell apoptosis.^20^ These differences in results may be attributable to the different mechanisms by which different sulfonylureas stimulate insulin secretion. Sulfonylureas block β‐cell ATP‐sensitive K^+^ channels, leading to depolarization of the cell membrane, opening of Ca^2+^ channels, and influx of Ca^2+^. These processes stimulate the secretion of insulin. Most sulfonylureas, particularly glibenclamide, non‐specifically affect SUR1 A and B and SUR2 that considerably increases Ca^2+^ influx and can induce apoptotic death of β cells.^16^ Gliclazide interacts specifically with the SUR1 site, increases the influx of Ca^2+^ to a much lesser extent, and thus prevents the induction of β‐cell apoptosis.

Gliclazide possesses antioxidant activity that can protect β cells from oxidative damage. In contrast to glibenclamide, gliclazide also suppresses the production of IL‐1‐β and TNF‐α in vitro and in vivo in mice and thus may have a protective effect on pancreatic cells by suppressing the production of these cytokines.^18^ However, an increase in β‐cell apoptosis during glibenclamide therapy was not observed in all reported studies. The *in vivo* effect of glibenclamide may cause a loss of the secretory ability of β cells as they become hyper‐excited but this secretory insufficiency is reversible. We assume that the loss of glycemic control and a marked decrease in insulin secretion during long‐term therapy with sulfonylureas results from impaired secretion and not an additional death of β cells.^6^ A decrease in the TV of β cells during therapy with sulfonylureas in our study probably occurred as a result of the depletion of β‐cells secretory function.

The effect of sulfonylureas on α cells has not been studied sufficiently. In addition, a comparative analysis of different sulfonylureas has not been conducted. Possibly, the development of chronic insulin hypersecretion during therapy with sulfonylureas leads to a decrease in the production of glucagon via paracrine regulation.^21^ Glucagon secretion increases when the function of β cells is depleted. This has been confirmed by the results of some studies: the concentration of glucagon in blood plasma did not change or decrease in patients without T2DM and with intact β‐cell function, but the concentration of glucagon increased in patients with β‐cell insufficiency after consuming both mixed food and glimepiride, regardless of the level of glucagon before exposure.^21^ Thus, we can assume that gliclazide reduces insulin secretion to a lesser extent. Gliclazide does not cause hypersecretion of glucagon by the paracrine mechanism and due to the decrease of glycemia reduces hyperplasia of α cells.

In our study, we also studied the effects of a combination of sulfonylureas with DPP4i. According to the literature, DPP4i reduces the production of proinflammatory cytokines. DPP4i probably affect the function and number of α and β cells to produce the same effect.^22^, ^23^, ^24^

According to the literature, DPP4i drugs regulate the proliferation and survival of pancreatic islet cells, increase their mass, stimulate the proliferation of β cells, and induce islet neogenesis.^25^

We have not found any literature data on the effect of the DPP4i‐therapy duration on the number of α cells in experiments using rats aged 12 months.

One of the mechanisms of the effect of DPP4i on endocrinocytes is probably a decrease in the production of proinflammatory cytokines. According to the literature, vildagliptin reduced the concentration of TNF‐α, IL‐6, markers of oxidative stress.^22^, ^23^, ^25^, ^26^ In particular, we suggest that a decrease in the production of proinflammatory cytokines during DPP4i therapy may be one of the reasons for the modulation of the number of α‐cells in our study.

An increase of β cells during DPP4i therapy was reported by A. S. Akarte (vildagliptin therapy for 8 weeks using an experimental model of type 2 diabetes in rats). Vildagliptin increased the expression level of GLP1 receptors in pancreatic β cells by 10‐fold compared with no treatment.

In addition, the following mechanism of action of vildagliptin is discussed. The ability of vildagliptin to initiate differentiation of mesenchymal stem cells (adipose tissue‐derived mesenchymal stem cells (ASCs)) derived from adipose tissue into insulin‐producing cells was demonstrated in a recent study. This was confirmed by morphological analysis and gene expression (PDX‐1, glucose transporter type 2 (Glut‐2), and insulin).^15^ This mechanism may also be related to the normalization of the β‐cells number seen in DPP4i therapy.

We assume that enhanced apoptosis of β cells does not occur during the development of T2DM. The process of dedifferentiation of β cells into β‐cell progenitors may potentially occur under conditions of hyperglycemia and other adverse factors. The process of re‐differentiation of β cells into other forms of pancreatic cells, in particular into α cells, also takes place. The number of β cells can be restored to some extent by the normalization of glycemia, weight, nutrition, as well as by the treatment with antidiabetic drugs.

Thus, it is possible that DPP4i drugs contribute to the recovery of β cells via differentiation processes and other factors. The results of our study indicate this to some extent. However, additional research with differentiation markers should be conducted to confirm our conclusions.

## 5 CONCLUSION

1. The TV of β and α cells in untreated rats with T2DM were similar to those in previous studies and differed significantly from those of the intact group.
2. DPP4i therapy for 24 weeks helped normalize the TV of β and α cells; no significant differences with the intact group were detected.
3. Neither glibenclamide monotherapy nor its combination with DPP4i normalized the TV of α cells. The TV of α cells in its groups was similar to that in rats with T2DM that did not receive treatment.
4. Normalization TV of α‐cell was observed with gliclazide monotherapy and its combination with DPP4i.
5. The addition of DPP4i to sulfonylureas normalized the TV of β‐cells.

## 6 LIMITATION

We did not determine the level of insulin and glucagon in rat blood. Such data would help reach better conclusions. Unfortunately, technical difficulties did not allow us to conduct these assessments.

## DISCLOSURES

The authors declare no conflict of interest.

## AUTHOR'S CONTRIBUTION

Research concept and design—Tuchina Taisiia Pavlovna, Babenko Alina Yurievna, Galagudza Mikhail Mikhailovich. Making an experiment—Tuchina Taisiia Pavlovna, Skotnikova Kseniia Petrovna, Vtorushina Anna Anatolievna, Uskov Ivan Sergeyevich. Data collection and processing—Tuchina Taisiia Pavlovna, Rogoza Olga Vladimirovna, Grozov Roman Viktorovich. Statistical processing—Tuchina Taisiia Pavlovna.; Writing the text of the article—Tuchina Taisiia Pavlovna.; Editing—Babenko Alina Yurievna, Galagudza Mikhail Mikhailovich. Approval of the final version of the article—all authors.

## Data Availability

The data that support the findings of this study are openly available in https://cloud.mail.ru/public/4ADj/4dcohXUt6
